# Sensitization and synergistic anti-cancer effects of Furanodiene identified in zebrafish models

**DOI:** 10.1038/s41598-019-40866-2

**Published:** 2019-03-14

**Authors:** Xiao-Yu Zhu, Dian-Wu Guo, Qiao-Cong Lao, Yi-Qiao Xu, Zhao-Ke Meng, Bo Xia, Hua Yang, Chun-Qi Li, Ping Li

**Affiliations:** 1 State Key Laboratory of Natural Medicines, China Pharmaceutical University, Nanjing, Jiangsu Province 210009 P. R. China; 2 Hunter Biotechnology, Inc, F1A, building 5, No. 88 Jiangling Road, Binjiang Zone, Hangzhou City, Zhejiang Province 310051 P. R. China; 3 Minsheng Biopharma Research Institute, F8, building F, No. 1378 Wenyixi Road, Yuhang Zone, Hangzhou City, Zhejiang Province 310011 P. R. China

## Abstract

Furanodiene is a natural terpenoid isolated from *Rhizoma Curcumae*, a well-known Chinese medicinal herb that presents anticancer effects in various types of cancer cell lines. In this study, we have successfully established zebrafish xenografts with 5 various human cancer cell lines; and validated these models with anti-cancer drugs used clinically for treating human cancer patients. We found that Furanodiene was therapeutically effective for human JF 305 pancreatic cancer cells and MCF-7 breast cancer cells xenotranplanted into zebrafish. Furanodiene showed a markedly synergistic anti-cancer effect when used in combination with 5-FU (5-Fluorouracil) for both human breast cancer MDA-MB-231 cells and human liver cancer BEL-7402 cells xenotransplanted into zebrafish. Unexpectedly, Furanodiene reversed multiple drug resistance in the zebrafish xenotransplanted with *cis-*Platinum-resistant human non-small cell lung cancer cells and Adriamycin-resistant human breast cancer cells. Furanodiene played its anti-cancer effects through anti-angiogenesis and inducing ROS production, DNA strand breaks and apoptosis. Furanodiene suppresseed efflux transporter Pgp (P-glycoprotein) function and reduced Pgp protein level, but no effect on Pgp related gene (*MDR1*) expression. These results suggest sensitizition and synergistic anti-cancer effects of Furanodiene that is worthy of a further investigation.

## Introduction

In recent years, the anti-cancer potential of natural products from medicinal plants, especially those used in traditional Chinese medicine, has been gradually recognized by the scientific community^1–5^. A few pure compounds isolated from traditional Chinese medicine, such as Berberine, Curcumin, Quercetin, and so on, have been studied to treat cancer in experimental and clinical investigations^6–8^.

*Curcumae Rhizoma* (*R. curcumae*), a commonly used traditional Chinese medicinal herb, has displayed a wide and diverse medicinal value for nearly one thousand years, including removal of blood stasis and pain alleviation. The herb has been widely prescribed to treat cardiovascular diseases and cancer in Chinese clinical practice^9^. Growing evidence suggests that *R. curcumae* extract has anti-proliferative and pro-apoptotic effects on various types of cancer cells^10–12^. Pharmacodynamic studies found that *R. curcumae* extracts, including curcumin and β-elemene, not only inhibit cancer cell growth^13^, but also demonstrate synergistic therapeutic effects with many chemotherapeutic agents such as 5-Fluorouracil (5-FU), Paclitaxel, Doxorubicin, and reduce the toxicity induced by anti-cancer drugs^14^.

Furanodiene is another sesquiterpene isolated from the essential oil of *Curcuma wenyujin*^15^, exhibiting hepatoprotective, anti-inflammatory, anti-angiogenic and anti-tumor activities^16–24^. *In vitro* studies suggested that Furanodiene enhanced the anti-cancer effects of doxorubicin on ERα-negative breast cancer cells^25^, presented synergistic anti-proliferative activity with paclitaxel via altering cell cycle and integrin signaling in 95-D lung cancer cells^26^, and combinational treatments with Furanodiene and Doxorubicin blocked the invasion and migration of MDA-MB-231 breast cancer cells *in vitro*^27^. However, the synergistic anti-cancer effects as well as probably sensitizing drugs-resistant cancer cells of Furanodiene have never been investigated in vivo in any animal cancer models.

Given the high genetic and physiological similarities with humans, zebrafish has been used as a powerful and cost-effective animal model for cancer research and cancer drug discovery^28–33^. Using zebrafish in cancer drug research and development provides several advantages including transparency, easy manipulation, high predictability, short testing period, and small amount of testing drugs required. Zebrafish cancer models could be established by carcinogen treatment, genetic knockout, gene overexpression or xenotransplanting human cancer cells into the zebrafish^33^. The translucent body of zebrafish enables researchers to visualize all processes associated with tumor formation, progression, metastasis and death^29–33^. The small molecule compounds or drugs can be added directly to the water environment of the zebrafish and the therapeutic effects could be assessed qualitatively and quantitatively.

In this study, we have successfully developed zebrafish xenografts with human A549 non-small-cell lung cancer cells, SGC-7901 stomach cancer cells, HepG2 liver cancer cells, JF 305 pancreatic cancer cells and MCF-7 breast cancer cells; and validated these models with anti-cancer drugs used clinically in treating cancer patients. We have also established zebrafish xenografts with 5 drug-resistant human gastric cancer cells. We found that the known anti-cancer agent Furanodiene isolated from traditional Chinese herbs are effective in treating human JF 305 pancreatic cancer cells and MCF-7 breast cancer cells xenotranplanted into the zebrafish. Furanodiene has clearly shown a synergistic anti-cancer effects when used in combination with 5-FU for both human breast cancer MDA-MB-231 cells and human liver cancer BEL-7402 cells xenotransplanted into the zebrafish. Surprisingly, we found that Furanodiene could reverse multiple drug resistance in zebrafish xenotransplanted with *cis-*Platinum-resistant human non-small cell lung cancer cells and Adriamycin-resistant human breast cancer cells. In the mechanistic studies, we have found that Furanodiene plays its anti-cancer roles through anti-angiogenesis and inducing ROS production, DNA strand breaks and apoptosis. We have also discovered that Furanodiene markedly suppresses efflux transporter P-glycoprotein (Pgp) function and reduces Pgp protein level, but no effect on Pgp related gene (*MDR1*) expression.

## Results

### Anti-cancer effects

To confirm whether response of zebrafish xenografts to cancer drugs is similar to the response of mammalian model systems, we treated the xenotransplanted zebrafish with each of 10 known anti-cancer drugs (Bevacizumab, *cis-*Platinum, Endostar, Paclitaxel, Vinorelbine, Adriamycin, 5-FU, Hydroxyurea, Irinotecan and Gemcitabine) and Furanodiene for 48 h. Three concentrations or dosages for each drug were assessed. The maximum tolerated concentration/dosages MTC/MTD of a testing drug was defined as a maximum concentration or maximum dosage that did not induce any observable adverse effect on zebrafish and was determined under a dissecting stereomicroscope by a well-trained zebrafish toxicologist. The MTC/MTD was 400 ng for Bevacizumab, 2 ng for *cis-*Platinum, 80 ng for Endostar, 2.56 ng for Paclitaxel, 0.5 ng for Vinorelbine, 2.5 ng for Adriamycin, 2 ng for Irinotecan, 20 ng for Gemcitabine, 260 μg/mL for 5-FU and 1000 μg/mL for Hydroxyurea, respectively and 12.2 ng for Furanodiene.

**Figure 1:**
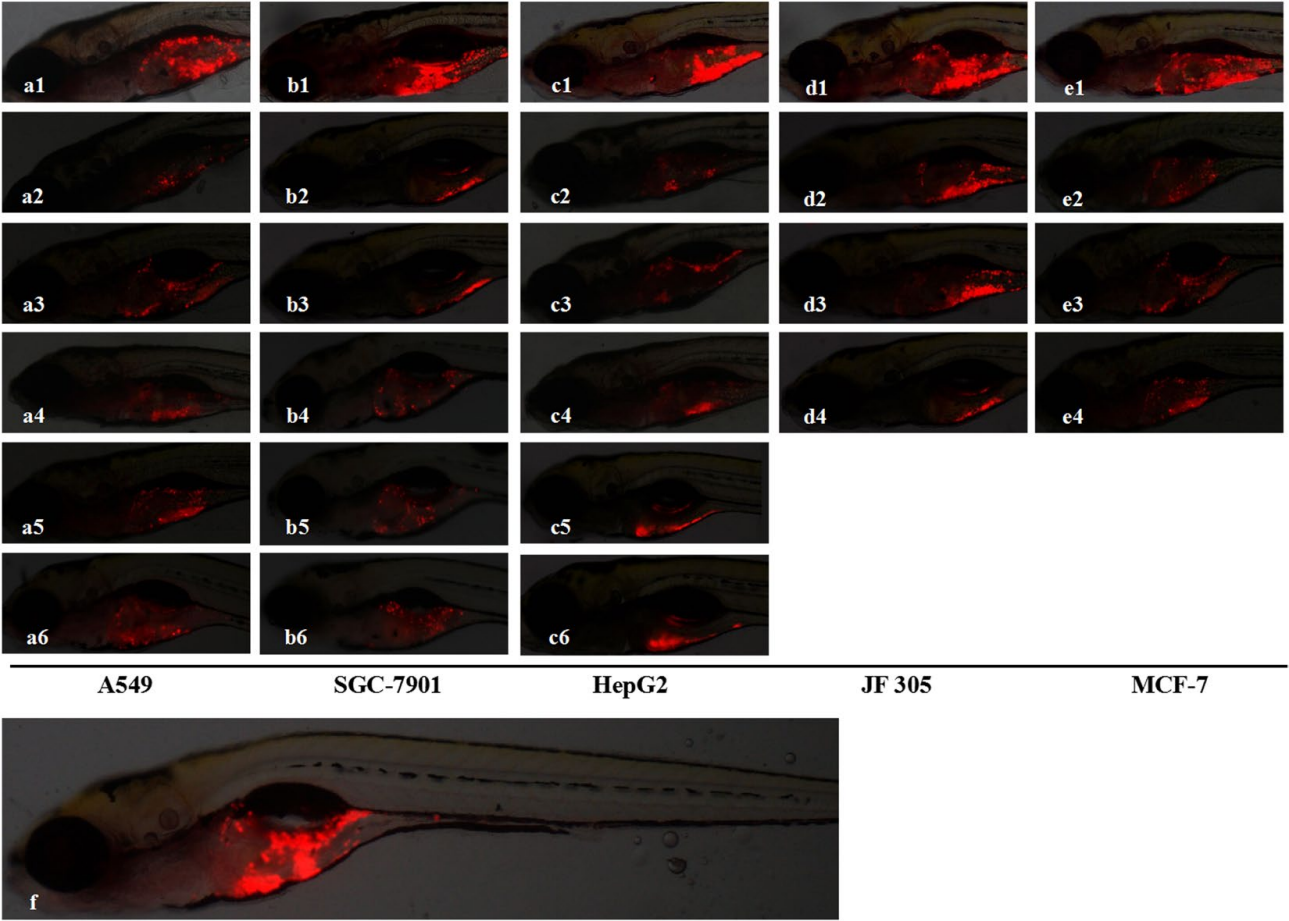
Cancer cell proliferations were suppressed after 48-h drug treatment in the zebrafish xenotransplanted with human A549 non-small-cell lung cancer cells, human SGC-7901 stomach cancer cells, human HepG2 liver cancer cells, human JF 305 pancreatic cancer cells, and human MCF-7 breast cancer cells. a1, b1, c1, d1 and e1 were the control xenotransplanted zebrafish without drug treatment. a2–a6 were treated with Bevacizumab, *cis*-Platinum, Endostar, Paclitaxel and Vinorelbine; b2–b6 were treated with 5-FU, Hydroxyurea, *cis*-Platinum, Irinotecan and Paclitaxel; c2–c6 were treated with Adriamycin, Gemcitabine, Hydroxyurea, *cis*-Platinum and 5-FU; and d2–d4 as well as e2–e4 were treated with Furanodiene at concentrations of 1/9 MTC, 1/3 MTC and MTC, respectively. A representative entire body of the xenotransplanted zebrafish body was shown in f.

**Figure 2:**
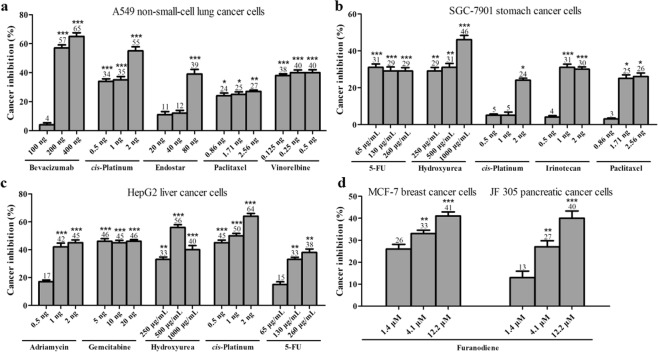
Quantitative analyses of cancer cell proliferations suppression in the zebrafish xenotransplanted with human A549 non-small-cell lung cancer cells (**a**) human SGC-7901 stomach cancer cells (**b**) human HepG2 liver cancer cells (**c**) human JF 305 pancreatic cancer cells and human MCF-7 breast cancer cells (**d**) after 48-h treatment of various anti-cancer drugs. As compared with untreated control xenotransplanted zebrafish, *p < 0.05, **p < 0.01, and ***p < 0.001.

Xenotransplanted zebrafish were generated by microinjection of approximately 800 human cancer cells labeled with CM-Dil into the yolk sac of zebrafish that were at 48 hpf as we reported previously^34^. As expected, after a 48 h treatment, Bevacizumab, *cis-*Platinum, Endostar, Paclitaxel, Vinorelbine, Adriamycin, 5-FU, Hydroxyurea, Irinotecan and Gemcitabine all significantly inhibit tumor growth in the zebrafish xenotransplanted with various types of human cancer cells (Fig. 1). The cancer inhibition percentages in zebrafish xenotransplant model of A549 non-small-cell lung cancer cells were (4 ± 1.39)–(65 ± 2.54)% for Bevacizumab, (34 ± 1.78)–(55 ± 2.89)% for *cis-*Platinum, (11 ± 2.13)–(39 ± 3.22)% for Endostar, (24 ± 1.99)–(27 ± 0.99)% for Paclitaxel, (38 ± 1.22)–(40 ± 2.01)% for Vinorelbine, respectively; in zebrafish xenotransplant model of SGC-7901 stomach cancer cells were (29 ± 1.87)–(31 ± 1.86)% for 5-FU, (29 ± 1.96)–(46 ± 2.31)% for Hydroxyurea, (5 ± 0.77)–(24 ± 1.11)% for *cis-*Platinum, (4 ± 0.89)–(31 ± 1.23)% for Irinotecan, (3 ± 0.56)–(26 ± 1.99)% for Paclitaxel, respectively; in zebrafish xenotransplant model of HepG2 liver cancer cells were (17 ± 1.22)–(45 ± 2.04)% for Adriamycin, (45 ± 1.94)–(46 ± 1.25)% for Gemcitabine, (33 ± 1.79)–(56 ± 1.96)% for Hydroxyurea, (45 ± 1.91)–(64 ± 2.08)% for *cis-*Platinum, (15 ± 1.95)–(38 ± 2.51)% for 5-FU, respectively. The cancer inhibition percentages for Furanodiene at 3 various concentrations were (13 ± 2.95)%, (27 ± 2.78)%, (40 ± 3.30)% in zebrafish xenotransplant model of JF 305 pancreatic cancer cells, and (26 ± 2.12)%, (33 ± 1.56)%, (41 ± 1.89)% in zebrafish xenotransplant model of MCF-7 breast cancer cells, respectively. Statistically significant inhibitory effects on zebrafish xenotransplants were observed for all the tested anticancer drugs and Furanodiene (p < 0.05 or p < 0.01 or p < 0.001) (Fig. 2).

The prolonged survival period was observed in the zebrafish xenotransplanted with HepG2 hepatocellular carcinoma cells and treated with Furanodiene. The survival days were extended for 1 time, 2 times and 2.67 times when treated with Furanodiene at 1/9 MTC, 1/3 MTC and MTC, respectively, as compared with untreated xenotransplanted zebrafish. Statistical differences (p < 0.05) were found in the zebrafish xenografs treated with Furanodiene at 1/3 MTC and MTC.

### Synergistic effects

**Figure 3:**
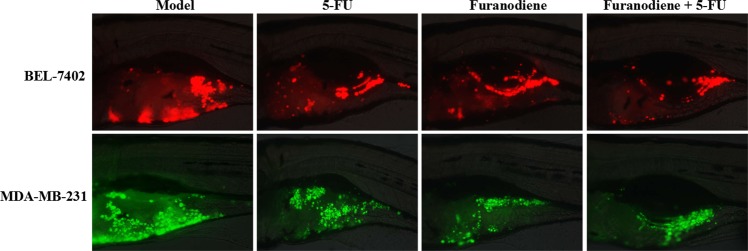
Synergistic anti-cancer effects of Furanodiene with 5-FU in zebrafish xenotransplanted with human BEL-7402 liver cancer cells (above) or with human MDA-MB-231 breast cancer cancer cells (below).

To determine whether Furanodiene had a synergistic effect, zebrafish at 48 hpf was xenotransplanted with human liver cancer BEL-7402 or breast cancer MDA-MB-231 sensitive cancer cell lines labeled with CM-Dil. The results showed that Furanodiene significantly enhance 5-FU inhibition of tumor growth on BEL-7402 and MDA-MB-231 sensitive cancer cell lines (Fig. 3, Table 1). The cancer inhibition % in zebrafish xenotransplanted with BEL-7402 was (34 ± 2.31)% for 5-FU, (23 ± 2.78)% for Furanodiene, (48 ± 3.26)% for the combination respectively; in zebrafish xenotransplant model of MDA-MB-231 was (35 ± 1.98)% for 5-FU, (5 ± 0.56)% for Furanodiene, and (45 ± 3.30)% for Furanodiene cotreatment with 5-FU, respectively. Statistical differences (p < 0.05 & p < 0.01) were found in the zebrafish xenografs treated with Furanodiene cotreatment with 5-FU as compared with 5-FU and Furanodiene alone.

**Table 1:** Synergistic anti-cancer effects of Furanodiene in zebrafish xenotransplant models (Mean ± SE).

Drugs	Concentration (μM)	Cancer inhibition on BEL-7402 (%)
5-FU	333	34 ± 2.31
Furanodiene	1.4	23 ± 2.78
Furanodiene + 5-FU	1.4 + 333	48 ± 3.26^#/##^
**Drugs**	**Concentration (μM)**	**Cancer inhibition on MDA-MB-231 (%)**
5-FU	333	35 ± 1.98
Furanodiene	1.4	5 ± 0.56
Furanodiene + 5-FU	1.4 + 333	45 ± 3.30^#/##^

#p < 0.05, as compared with 5-FU; and ##p < 0.01, as compared with Furanodiene.

### Anti-cancer sensitization effects

**Figure 4:**
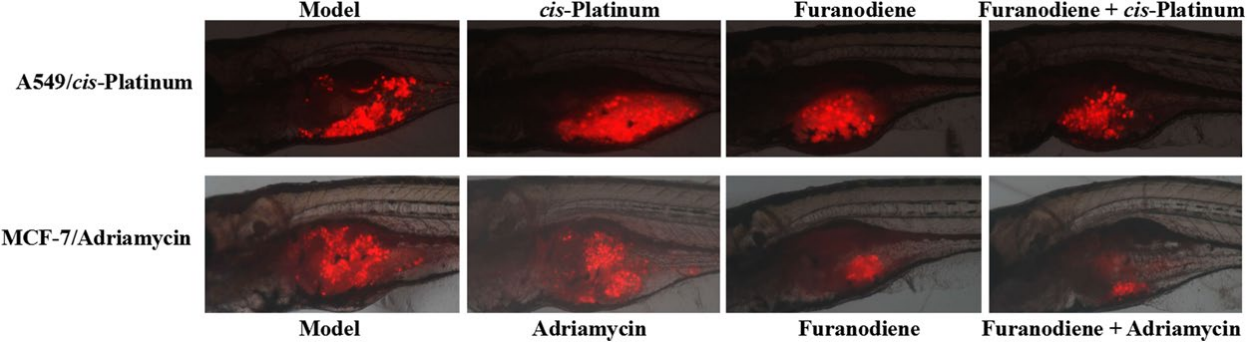
Sensitizing effect of Furanodiene on *cis*-Platinum-resistant human A549 non-small-cell lung cancer cells (above) and Adriamycin-resistant MCF-7 breast cancer cells (below) xenotransplanted into zebrafish.

Zebrafish xenotransplanted with drug-resistance human cancer cells were used to assess anti-multidrug resistance effects of Furanodiene. We have successfully established 5 zebrafish xenotransplant models for anti-multidrug resistance assessment, including the zebrafish xenotransplanted with *cis*-Platinum-resistant human gastric cancer cells, *cis*-Platinum-resistant human non-small cell lung cancer cells, Adriamycin-resistant human breast cancer cells, paclitaxel-resistant human ovarian cancer cells, and Adriamycin-resistant human oral squamous cancer cells. To determine whether Furanodiene could reverse multidrug resistance, zebrafish at 48 hpf was xenotransplanted approximately 800 human cancer drug resistant cells (A549/*cis-*Platinum and MCF-7/Adriamycin) labeled with CM-Dil. The results showed that *cis-*Platinum or Adriamycin had no statistically effect on the zebrafish xenotransplanted with human cancer drug resistant cells (A549/*cis-*Platinum and MCF-7/Adriamycin); Furanodiene and Furanodiene cotreatment with *cis-*Platinum or Adriamycin significantly inhibit tumor growth in zebrafish xenotransplanted with human cancer drug resistant cells (A549/*cis-*Platinum and MCF-7/Adriamycin) (Fig. 4). The cancer inhibition percentages in zebrafish xenotransplant model of A549/*cis-*Platinum were (2 ± 1.68)–(28 ± 1.85)% for Furanodiene, (21 ± 2.03)–(41 ± 3.41)% for Furanodiene cotreatment with *cis-*Platinum, respectively; in zebrafish xenotransplant model of MCF-7/Adriamycin were (11 ± 1.20)–(40 ± 2.23)% for Furanodiene, (27 ± 2.56)–(59 ± 4.20)% for Furanodiene cotreatment with Adriamycin, respectively (Table 2). Statistically significant inhibitory effects on zebrafish xenotransplants with human cancer drug resistant cells (A549/*cis-*Platinum and MCF-7/Adriamycin) were observed for Furanodiene (p < 0.05 or p < 0.01 or p < 0.001), and statistical differences (p < 0.001) were found in the zebrafish xenografs treated with Furanodiene cotreatment with *cis-*Platinum/Adriamycin as compared with *cis*-Platinum/Adriamycin alone.

**Table 2:** Sensitizing effect of Furanodiene on zebrafish xenografts (Mean ± SE).

Drugs	Concentration (μM)	Cancer inhibition on A549/cis-platinum (%)
cis-Platinum	100	−2 ± 0.99
Furanodiene	1.4	2 ± 1.68
	4.1	16 ± 3.26*
	12.2	28 ± 1.85***
Furanodiene + *cis*-Platinum	1.4	21 ± 2.03*###
	4.1	39 ± 3.26**###
	12.2	41 ± 3.41***###
**Drugs**	**Concentration (μM)**	**Cancer inhibition on** **MCF-7/adriamycin (%)**
Adriamycin	10 µg/mL	−0.1 ± 0.39
Furanodiene	1.4	11 ± 1.20
	4.1	14 ± 2.01*
	12.2	40 ± 2.23***
Furanodiene + Adriamycin	1.4	27 ± 2.56***###
	4.1	36 ± 3.01***###
	12.2	59 ± 4.20***###

Compared with control: *p < 0.05, **p < 0.01 & ***p < 0.001.

Furanodiene cotreat compared with a tested drug alone: ^#^p < 0.05, ^##^p < 0.01, and ^###^p < 0.001.

### Mechanisms

#### Antiangiogenesis

Antiangiogenic effect of Furanodiene was assessed on 2 dpf Tg (fli1a: EGFP) transgenic zebrafish with fluorescent blood vessels. In the initial pilot study, we found the growth of angiogenic SIVs were markedly inhibited in zebrafish treated with Furanodiene at a concentration of MTC for 24 h. Some of the SIVs were incompletely formed resulting in a significant reduction in the total area of SIVs, as compared with untreated control zebrafish. In the dose-response experiments, the SIVs inhibition % of Furanodiene at 1/9 MTC, 1/3 MTC and MTC on angiogenesis was (19 ± 2.19)%, (21 ± 2.33)% and (43 ± 3.55)%, respectively (Table 3). Statistically significant differences (p < 0.05 & p < 0.01) were found in zebrafish treated with Furanodiene at 1/3 MTC and MTC.

**Table 3:** Mechanism study of Furanodiene in zebrafish models (Mean ± SE).

Assays	Biomarkers	Efficacy (%)		
		1.4 μM	4.1 μM	12.2 μM
Antiangiogenesis	SIVs inhibition	19 ± 2.19	21 ± 2.33^*^	43 ± 3.55^**^
Apoptosis	AO staining	20 ± 3.29	51 ± 2.31^***^	65 ± 0.99^***^
	caspase-3/7	2 ± 1.78	16 ± 1.47^*^	67 ± 1.66^***^
	caspase-8	11 ± 1.56	17 ± 1.46^*^	68 ± 2.17^***^
	caspase-9	8 ± 1.69	17 ± 2.31^*^	69 ± 2.85^***^
Comet assay	Single stranded DNA strand fracture analysis	116 ± 3.58	370 ± 2.45^**^	540 ± 3.97^***^
	Double stranded DNA strand fracture analysis	118 ± 2.79	143 ± 3.12^**^	208 ± 2.25^***^
ROS	ROS production	145 ± 1.56	190 ± 3.18^*^	521 ± 3.98^***^
P-glycoprotein	Pgp inhibition	21 ± 1.88^*^	34 ± 2.05^*^	58 ± 1.82^*^

Compared with control: *p < 0.05, **p < 0.01 and ***p < 0.001.

#### ROS, DNA strand breaks and apoptosis

ROS levels in zebrafish treated with Furanodiene were analyzed using an oxidation sensitive probe, 5-(and 6-)-chloromethyl-20, 70-dichloro-dihydrofluoresceindiacetate (CM-H2DCFDA, Life Technologies, Carlsbad, CA). Furanodiene treatment resulted in increased ROS production. ROS levels relative to untreated control zebrafiash were (145 ± 1.56)%, (190 ± 3.18)% and (521 ± 3.98)%, respectively, in zebrafish treated with Furanodiene at concentrations of 1/9 MTC, 1/3MTC and MTC (Table 3). Statistically significant differences (p < 0.05 & p < 0.001) were found in zebrafish treated with Furanodiene at 1/3 MTC and MTC.

**Figure 5:**
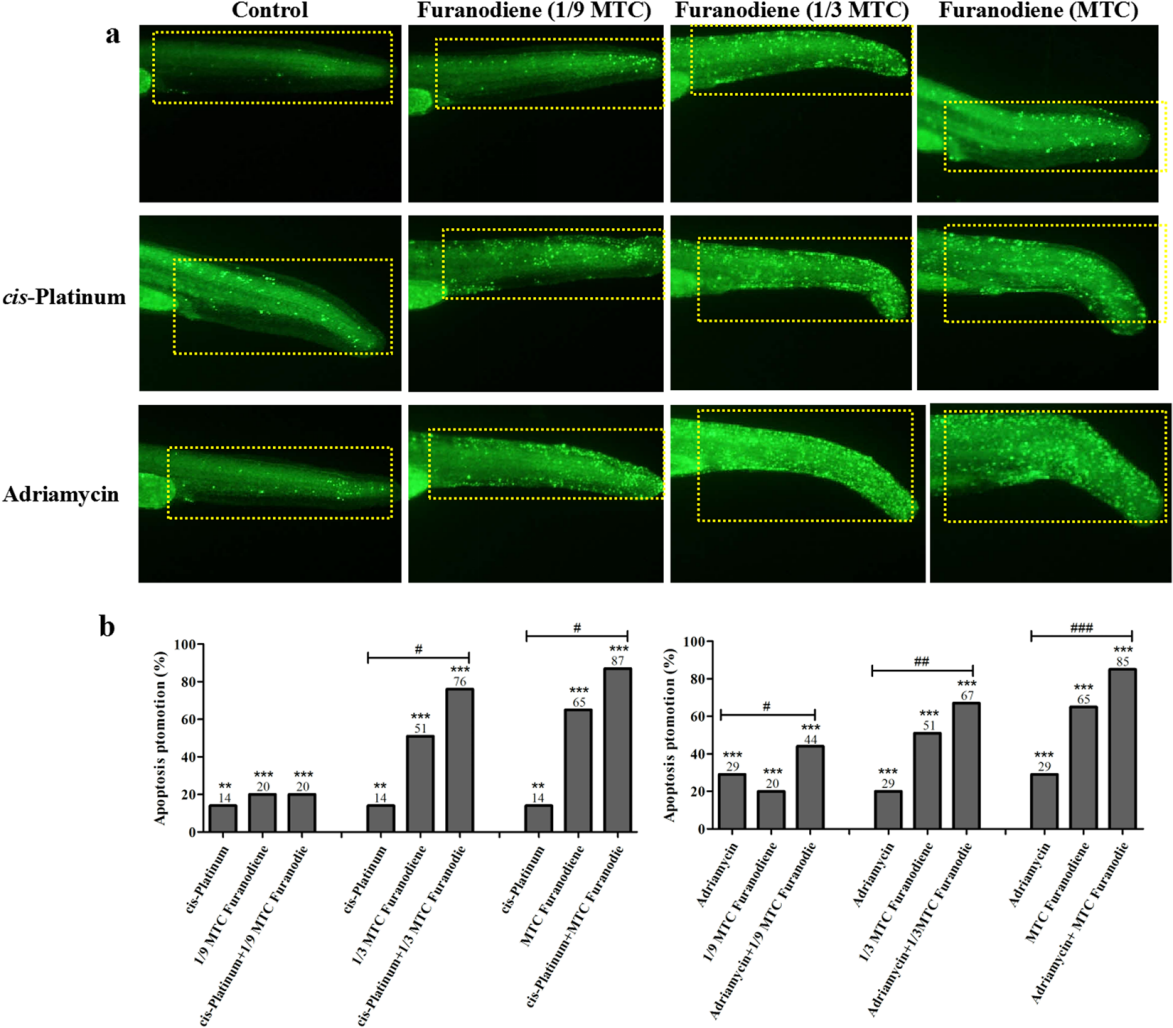
Apoptosis in zebrafish treated with *cis*-Platinum or Adriamycin alone, and *cis*-Platinum or Adriamycin in combination with Furanodiene at 1/9 MTC, 1/3 MTC and MTC, respectively (**a**). Quantitative data of (**a**) were shown in (**b**).

Single strand DNA breaks in the zebrafish cells treated with 1/9 MTC, 1/3 MTC and MTC of Furanodiene were (116 ± 3.58)%, (370 ± 2.45)% and (540 ± 3.97)% higher than in untreated control zebrafish cells. Double DNA breaks were elevated to (118 ± 2.79)%, (143 ± 3.12)% and (208 ± 2.25)% relative to untreated control zebrafish (Table 3). Noteworthy differences (p < 0.01 & p < 0.001) in both single and double DNA breaks were observed in zebrafish treated with Furanodiene at 1/3 MTC and MTC (Fig. 5, Table 3).

**Figure 6:**
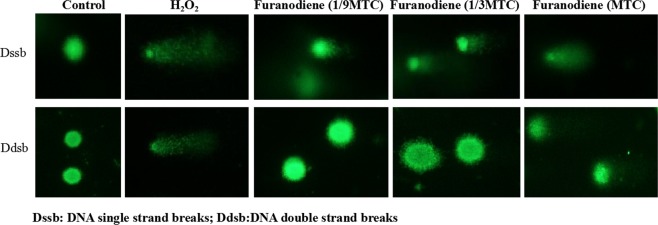
DNA single strand breaks (above) and DNA double strand breaks (below) in zebrafish treated with Furanodiene at 1/9 MTC, 1/3 MTC and MTC. H_2_O_2_ was used as a positive control.

The tailbud of zebrafish was chosen for the observation of apoptosis induction^35^. Zebrafish treated with 0.1% DMSO had a few apoptotic cells, while *cis-*Platinum treatment significantly increased the number of apoptotic cells. When treated with different concentrations of Furanodiene, apoptosis were increased at a dose-dependent manner. The promotion percentage on apoptosis was (14 ± 2.38)% for *cis-*Platinum at 100 μM, (29 ± 2.65)% for Adriamycin at 10 µg/mL, (20 ± 3.29)–(65 ± 1.99)% for Furanodiene at 1/9 MTC, 1/3 MTC and MTC, (20 ± 2.59)–(87 ± 1.49)% for Furanodiene cotreatment with *cis-*Platinum, and (44 ± 3.15)–(85 ± 2.49)% for Furanodiene cotreatment with Adriamycin, respectively (Fig. 6). Statistically significant (p < 0.01 & p < 0.001) promotion effects on apoptosis were observed for Furanodiene, Adriamycin, *cis-*Platinum, Furanodiene cotreatment with Adriamycin/*cis-*Platinum, Statistical differences (p < 0.05 & p < 0.01 & p < 0.001) were found in the zebrafish treated with Furanodiene cotreatment with *cis-*Platinum/Adriamycin as compared with *cis-*Platinum/Adriamycin alone. Caspase activities were measured using caspase-Glo reagents (Promega, USA) through cleavage of colorless substrates specific for caspase 3/7 caspase 8 and caspase 9. As indicated in Table 3, after treatment with Furanodiene at 1/9 MTC, 1/3 MTC and MTC, caspase-3/7, -8 and -9 activities were all notably increased by (2 ± 1.78)%, (16 ± 1.47)% and (67 ± 1.66)%; (11 ± 1.56)%, (17 ± 1.46)% and (68 ± 2.17)%; and (8 ± 1.69)%, (17 ± 2.31)% and (69 ± 2.85)%, respectively. Statistically significant differences (p < 0.05 & p < 0.001) for caspase 3/7 caspase 8 and caspase 9 were all found in zebrafish treated with Furanodiene at 1/3 MTC and MTC.

#### Pgp efflux regulation

**Figure 7:**
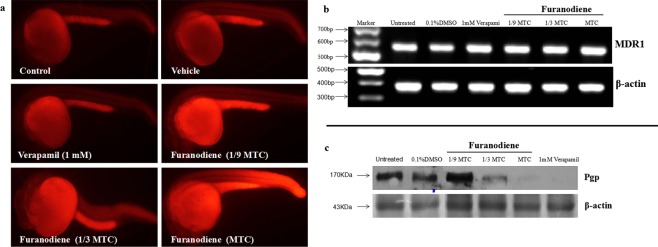
Pgp efflux transporter regulation in zebrafish treated with Furanodiene. (**a**) Pgp function inhibition by Furanodiene at 1/9 MTC, 1/3 MTC and MTC, 1 mM Verapamil was used as a positive control; (**b**) Pgp gene expression analyses by PCR; and (**c**) Pgp protein assay by Western blot.

Based on retention of rhodamine 123 in the whole zebrafish, we developed and validated an *in vivo* assay for assessing drug effects on Pgp efflux in zebrafish (our published patent: China patent No. 201110231726.2). As shown in Fig. 7, fluorescence signal was absent in control group, indicating that the dye was pumped out of zebrafish; while was retained in zebrafish after treatment with Furanodiene. The Pgp inhibition percentage of Furanodiene at 3 various concentrations (1/9 MTC, 1/3 MTC and MTC) was (21 ± 1.88)%, (34 ± 2.05)% and (58 ± 1.82)%, respectively (Table 3). Statistically significant differences were found in zebrafish treated with Furanodiene at 1/9 MTC (p < 0.05), 1/3 MTC (p < 0.05) and MTC (p < 0.05).

The mRNA levels of the Pgp related gene (*MDR1*) was determined in zebrafish treated with Furanodiene. The relative expression level of *MDR1* gene was unchanged in zebrafish treated with Furanodiene at concentrations of 1/3 MTC and MTC as compared with control group, whereas the expression level of Pgp protein was severely decreased.

## Dissussion

In this study, we have demonstrated that all the human anti-cancer drugs currently used clinically for cancer patients and selected for this study are therapeutically effective on the human cancer cells xenotranplanted into zebrafish, further validating and supporting zebrafish xenografts as a rapid, reliable, reproducible, and cost-efficient animal model for anti-cancer drug screening and assessment^34–39^. Our results from this investigation have confirmed that zebrafish xenotransplanted cancer models are also suitable for assessing sensitization and synergistic effects of anti-cancer drugs.

Furanodiene has been extensively studied as an anti-cancer agent in a variety of *in vitro* and *in vivo* models^19–27^. Here we have indicated that Furanodiene are effective for two types of human cancer cells (JF 305 pancreatic cancer cells and MCF-7 breast cancer cells) xenotranplanted into zebrafish, supporting that Furanodiene could be a potent anti-cancer drug^20–27^. Interestingly, in this study, Furanodiene has shown statistically significant synergistic anti-cancer effects when used in combination with 5-FU for both human breast cancer MDA-MB-231 cells and human liver cancer BEL-7402 cells xenotransplanted into the zebrafish.

In the mechanistic studies, we have found that Furanodiene plays its anti-cancer role through anti-angiogenesis, inducing ROS production, DNA strand breaks and apoptosis. Neoangiogenesis has been recognized as a hallmark of tumor progression and about a dozen of anti-angiogenetic drugs have been globally marked^40–44^. Elevated ROS production has been indicated to damage large biological molecules including DNA, leading to DNA strand breaks and apoptosis^45–47^. Caspase 8 participates in the intracellular signaling cascade leading to apoptosis while caspase 9 has been linked to the mitochondrial death pathway^48–51^. Caspase 3/7 is either partially or totally responsible for the proteolytic cleavage of many key proteins during apoptosis and interacts with caspase 8 and caspase 9^48–51^. Our results showed that the activity of caspases 8, 9 and 3/7 were all notablely increased in the cancer cell xenotransplanted zebrafish treated with Furanodiene, revealing that Furanodiene-induced zebrafish apoptosis are both caspase 8 and caspase 9 dependent, leading to cancer cell death.

Multiple-drug resistance is a most common and challengable problem in cancer treatment for both cytotoxic and targeted anti-cancer drugs^52^ and there are no effective sensitizing drugs available in market^53^. Surprisingly, in this study, when *cis-*Platinum-resistant human non-small cell lung cancer cells and Adriamycin-resistant human breast cancer cells xenotransplanted into zebrafish are co-treated with Furanodiene, significant cancer suppression is demonstrated that are statistically more potent than either *cis-*Platinum/Adriamycin (no significant inhibition) or Furanodiene alone. We have also discovered that Furanodiene markedly suppresses efflux transporter Pgp function and reduces Pgp protein, but no effect on *Pgp* gene expression. These data suggest that Furanodiene could be a potential sensitizing agent to anti-cancer drugs probably mainly through blocking Pgp efflux transportation. These results are consistant with other reports indicating that Pgp is one of major players responsible for anti-cancer drug resistance and blocking Pgp could reverse the cancer drug resistence^54–57^, Further studies are under progresses to confirm Furanodiene pharmacology in mammalian models and to investigate other cellular, biochemical, and molecular mechanisms involved in Furanodiene anti-cancer pathways.

## Materials and Methods

### Zebrafish care and maintenance

Two lines of zebrafish were used in this study: wild-type AB line and Tg (fli1a: EGFP) y1 zebrafish. Adult zebrafish were housed and maintained in accordance with standard procedures. Zebrafish were generated by natural pair-wise mating according to the Zebrafish Handbook^34^. Four to five pairs of adult zebrafish were set up for each mating, and on average 200–300 zebrafish per pair were generated. Zebrafish were maintained at 28 °C in fish water (0.2% Instant Ocean Salt in deionized water, pH 6.9–7.2, conductivity 480–510 μS/cm, and hardness 53.7–71.6 mg/L CaCO_3_). Zebrafish were cleaned and staged at 6 and 24 hours post fertilization (hpf)^34^. The University Animal Care and Use Committee approved of the animal procedures described here. These procedures are consistent with the American Veterinary Medical Association’s (AVMA) Panel on Euthanasia. All the zebrafish experiments were performed and completed at Hunter Biotechnology, Inc. and its zebrafish facility is accredited by the Association for Assessment and Accreditation of Laboratory Animal Care (AAALAC) International^34^.

### Chemicals and compounds

Endostar (lot #: 201505012) was purchased from Shandong Xiansheng Madejin Bio-Pharmaceuticals, China. Paclitaxel (lot #: 45076), *cis-*Platinum (lot #: k1520124) and Adriamycin (lot #: 25316-40-9) were purchased from Aladdin company of Shanghai, China. Bevacizumab (lot #: H0126805) was bought from Roche, Switzerland. Vinorelbine (lot #: 140501) was purchased from Jiangsu Haosen Pharmaceuticals. CM-DiI (lot #: V22888) was bought from Molecular Probes, USA. All the other agents were bought from Sigma-Aldrich. Cancer cells were purchased from American Type Culture Collection (ATCC).

### Determination of maximum tolerated concentration/dosages (MTC/MTD)

To determine MTC/MTD of a testing drug, zebrafish were treated with a testing drug and mortality and toxicity were recorded at the end of treatment. In the initial tests, five concentrations (0.1, 1, 10, 100, and 500 μg/mL for soaking drugs and 0.1, 1, 10, 100, and 500 ng for injection drugs) were used for each drug. If a MTC/MTD could not be found from the initial tests, additional concentrations within the range of 0.01–2000 μg/mL or 0.01–2000 ng were tested. The MTC/MTD of a testing drug was defined as a maximum concentration or maximum dosage that did not induce any observable adverse effect on zebrafish and was determined under a dissecting stereomicroscope by a well-trained zebrafish toxicologist.

### Cell culture

Human cancer cells were purchased from American Type Culture Collection (ATCC) and were subcultured and maintained in tissue culture flasks at 37 °C in a humidified, 5% CO2 atmosphere in RPMI 1640 essential medium or in DMEM essential medium (Gibco), supplemented with 10% heat-inactivated fetal bovine serum, 100 units/ml penicillin, 100 g/ml streptomycin, and 2 mM L-glutamine (Gibco)^34^.

### Cell labeling

Human cancer cells were collected by centrifugation and resuspended in phosphate-buffered saline (PBS). The cells were fluorescently labeled by incubating with 10 µg/ml CM-DiI (Invitrogen, Burlington, ON, Canada) containing 0.5% DMSO and were adjusted to a density of 100 × 10^6^ cells/ml (100 cells per nL) in HBSS. Labeled cells were injected within 2 hours. Cells prepared for injection in this way routinely had fewer than 5% dead cells and were in a single cell suspension, yielding controllable and reproducible numbers of injectable cells that were highly and uniformly fluorescent. The dye was transferred from mother to daughter cell and fluorescent single cells were clearly visible after several doublings on the periphery of late arising tumors^34^.

### Cell transplantation

The transplantation protocol was similar to that described by our previous report^34^. CM-DiI-labeled human cancer cells were loaded into a pulled glass micropipette (VWR blood capillaries #53508-400) that was drawn on an electrode puller and then trimmed to form a needle with a resulting internal diameter of approximately 15 micron and outer diameter of approximately 18 micron. The microneedle was attached to an air driven Cell Tram (Eppendorf). The tip of the needle was inserted into the yolk of a 48 hpf zebrafish and the pulse time controlled to deliver ~500 cells in 15 nL using positive pressure. The number of injected cells was standardized by fixing cell density and injection volume. After one hour recovery period at 28 °C, implanted zebrafish were examined under a fluorescence microscope (AZ100, Nikon, Japan) for the presence of xenotransplanted cells that reside only in the yolk and are then transferred to 35 °C for the duration of the experiment^34^.

### Assessment of anti-cancer effects

Xenotransplanted zebrafish were generated by microinjection of approximately 800 human cancer cells labeled with CM-Dil into the yolk sac of zebrafish that were at 48 hpf as we reported previously^34,36,37^. At 24 h post-xenotransplantation (hpx), drugs at 3 various concentrations (1/9 MTC, 1/3 MTC and MTC or 1/4 MTC, 1/2 MTC and MTC) was added to the treatment solution (fish water) for a drug treatment period of 72 hours (h). The xenotransplanted zebrafish treated with 0.1% DMSO was used as the cancer model group. After treatment, xenotransplanted zebrafish were photographed under a fluorescence microscope. Nikon NIS-Elements D 3.10 software was used to quantify all the cancer cell fluorescence intensity (S) in xenotransplanted zebrafish. Drug effect was calculated using the following formula: The cancer inhibition % = (1 − S(Drug)/S(Control)) × 100%.

### Determination of prolonged survival period

Xenotransplanted zebrafish were used to determine effects of drugs on the survival period of xenografted zebrafish. At 24 hpx xenotransplanted zebrafish were treated with a testing drug at 3 various concentrations (1/9 MTC, 1/3 MTC and MTC) for a treatment period of 9 days (d). The number of dead zebrafish in each group was recorded on a daily basis. Prolonged survival period was calculated based on the survival rate of xenotransplanted zebrafish treated with tested agents, as compared with untreated cancer model group. The prolonged survival period was calculated using the following formula: The prolonged survival period time = S(Drug)/S(Control).

### Quanlification of synergistic anti-cancer effects

To determine whether Furanodiene had a synergistic effect, zebrafish at 48 hpf was xenotransplanted with human liver cancer BEL-7402 or breast cancer MDA-MB-231 sensitive cancer cell lines labeled with CM-Dil. At 24 hpx, 5-Fluorouracil (5-FU) at 1/3 MTC, Furanodiene at 1/9 MTC, and Furanodiene at 1/9 MTC in combination with 5-FU at 1/3 MTC, respectively, were added to the treatment solution for a treatment period of 72 h. After treatment, xenotransplanted zebrafish were photographed under a fluorescence microscope and fluorescence intensity from the xenotransplanted cancer cells was quantified as described above^35,37–39^.

### Measurement of anti-cancer sensitization

Zebrafish xenotransplanted with drug-resistance human cancer cells were used to assess anti-multidrug resistance effects of Furanodiene. At 24 hpx, Furanodiene at 3 various concentrations (1/9 MTC, 1/3 MTC and MTC), chemotherapeutic drugs (cis-Platinum and Adriamycin) at MTC, Furanodiene at 3 various concentrations (1/9 MTC, 1/3 MTC and MTC) cotreated with a chemotherapeutic drug at MTC were added to the treatment solution for a treatment period of 72 h. Xenotransplanted zebrafish were photographed and fluorescence intensity was quantified.

### Mechanism study

#### Antiangiogenesis assessment

Tg (fli1a: EGFP) zebrafish at 48 hpf were distributed into 6-well microplates (Nest, NEST Biotech), 30 zebrafish per well in 3 mL fish water. Furanodiene at 3 various concentrations (1/9 MTC, 1/3 MTC and MTC) were added to the treatment solution for treatment period of 24 h. After treatment, zebrafish were anesthetized with 0.016% MS222 (tricaine methanesulfonate, Sigma-Aldrich, St. Louis, MO), then the number of ISVs was counted and the area of SIVs were quantified with NIS-Elements D3.1 software as we reported previously^37^.

#### Reactive oxygen species (ROS) measurement

ROS levels in zebrafish treated with Furanodiene were analyzed using an oxidation sensitive probe, 5-(and 6-)-chloromethyl-20, 70-dichloro-dihydrofluoresceindiacetate (CM-H2DCFDA, Life Technologies, Carlsbad, CA). The treated zebrafish were incubated with 0.5 mg/mL CM-H2DCFDA for 1 h in dark at 28 °C. After rinsing for 3 times using fish water, zebrafish were transferred into a 96-well microplate (1 zebrafish per well) and ROS was measured at 488 nm under a multimode microplate reader (Berthold Technologies, Mithras LB940, Germany) as described by our group^39^.

#### DNA strand break analysis

After treatment, zebrafish were lyzed into a single cell suspension. Zebrafish cells were combined with agarose at 1:10 ratio (v/v), mixed well by pipetting and immediately transferred onto the slide. The slides were carefully transferred from either neutral solution (for double DNA strand break detection) or alkaline solution (for single DNA strand break detection) to a horizontal electrophoresis chamber and electrophoresed at 28 volts for 20 min. DNA Staining was done by Vista Green DNA Dye (Cell Biolabs, San Diego, USA) and the Olive tail moment (OTM) was utilized as a biomarker to quantify DNA strand as reported by one of our co-authors^58^.

### Apoptosis quantifications

#### Acridine orange staining

AB wild type zebrafish at 6 hpf were distributed into 6-well microplates, 30 zebrafish per well in 3 mL fish water. Furanodiene at 3 various concentrations (1/9 MTC, 1/3 MTC and MTC) were added to the treatment solution for treatment period of 24 h. After treatment, zebrafish were stained with acridine orange and observed for apoptotic cells that would display yellow-green fluorescent spots under the fluorescence microscope. Nikon NIS-Elements D 3.10 Advanced image processing software was used to capture and analyze the images. The fluorescence signal from apoptotic cells was measured and the apoptotic rate was calculated as reported by us^58^.

#### Caspase activity assay

After treatment, caspase activities were measured using caspase-Glo reagents (Promega, USA) through cleavage of colorless substrates specific for caspase 3/7 caspase 8 and caspase 9 using a multifunction microplate reader (Berthold Technologies, Mithras LB940, Germany). In each assay, at least six wells per sample were measured for each dose and the results were averaged^59^.

### Pgp assays

#### Functional analysis

Based on retention of rhodamine 123 in the whole zebrafish, we developed an *in vivo* assay for assessing drug effects on Pgp efflux in zebrafish (our published patent: China patent No. 201110231726.2). In these studies, we determined that rhodamine 123 staining for 40 min was the optimal time point for assessing Pgp efflux in untreated and drug treated animals. AB wild type zebrafish at 6 hpf were distributed into 6-well microplates (Nest, NEST Biotech), 30 zebrafish per well in 3 mL fish water. Furanodiene at 3 various concentrations (1/9 MTC, 1/3 MTC and MTC) was added to the treatment solution. After 24 h treatment, zebrafish were stained with rhodamine 123 for 40 min, then photographed under a fluorescence microscope. Nikon NIS-Elements D 3.10 software was used to quantify the whole zebrafish fluorescence intensity.

#### Western blot

Total protein of compound-treated zebrafish was extracted with the Tissue Protein Rapid Extraction Kit (Keygen, China) and determined by performing the bicinchoninic acid (BCA) protein assay (Pierce, Waltham, MA). The equal amounts of 100 mg lysate proteins were loaded onto SDS-polyacrylamide gels (12%) at 80 V and electrophoretically transferred onto polyvinylidene difluoride membranes (Millipore, Billerica, MA) in Tris-glycine buffer at 100 V in an ice box. After blocking with 5% nonfat milk in TBST for 1 h at room temperature, the membrane was incubated with TNNT2 (1:1000, rabbit antibodies, Abcam, London, UK) overnight at 4 °C, After washed thrice with TBST, the membrane was incubated with a horseradish peroxidase-conjugated anti-rabbit Ig G secondary antibody (Abcam, Britain) for 1 h on a shaking table at room temperature, and washed thrice with TBST and the antibody-bound proteins were detected using the ECL chemiluminescence reagent (Pierce, Waltham, MA).

#### Quantitative RT-PCR

After treatment, total RNA of the treated zebrafish was isolated using TRIzol reagent (Invitrogen, Carlsbad, CA, SA). First-strand cDNA was reversely transcribed from total RNA using a QuantScript RT Kit (TIANGEN, Beijing, China) with Quant Reverse Transcriptase according to the manufacturer’s instruction. The primers were as follows, β-actin: 5′-CATCAGCATGGCTTCTGCTCTGTATGG-3′ and 5′-GACTTGTCAG TGTACAGAGACACCCT-3′; *MDR1*: 5′-CCTGGCTTCTCAATCTCAT- 3′ and 5′-TTTAC TACTCCCTTGTAACGC-3′. The expression of selected genes was analyzed based on the optical density (OD) of the corresponding bands with β-actin as the internal reference.

#### Statistical analysis

All data were presented as mean ± SE. Statistical analysis and graphical representation ofthe data were performed using GraphPad Prism 5.0 (GraphPad Software, San Diego, CA). If a single concentration of a drug was assessed, the Student’s t-test was used to identify drugs that exhibit a significant effect compared to the vehicle control group. If multiple concentrations of a drug were assessed, ANOVA was first used to assess whether there were any differences in the mean among the various concentrations of each compound; if a significant difference was determined (p < 0.05), Dunnett’s test, which is appropriate for multiple pair-wise comparisons against a control, was then performed.

## Data Availability

The datasets generated and/or analysed during the current study are available from the corresponding author on reasonable request.
